# From simple to even simpler, but not too simple: a head-to-head comparison of the Better-Worse and Drop-Down methods for measuring patient health status

**DOI:** 10.1186/s12874-023-02119-9

**Published:** 2023-12-16

**Authors:** Xin Zhang, Paul F. M. Krabbe

**Affiliations:** Department of Epidemiology, University Medical Center Groningen, University of Groningen, Hanzeplein 1, Groningen, 9713 GZ The Netherlands

**Keywords:** Patient-reported outcome measures (PROMs), Health status, Health-related quality of life (HRQoL), Measurement model, Preference-based, Values

## Abstract

**Background:**

We recently developed a novel, preference-based method (Better-Worse, BW) for measuring health status, expressed as a single metric value. We have since expanded it by developing the Drop-Down (DD) method. This article presents a head-to-head comparison of these two methods. We explored user feasibility, interpretability and statistics of the estimated coefficients, and distribution of the computed health-state values.

**Methods:**

We conducted a cross-sectional online survey among patients with various diseases in the USA. The BW and DD methods were applied in the two arms of the study, albeit in reverse order. In both arms, patients first performed a descriptive task (Task 1) to rate their own health status according to the 12 items (each with 4 levels) in the CS-Base health-outcome instrument. They then performed Task 2, in which they expressed preferences for health states by the two methods. We then estimated coefficients for all levels of each item using logistic regression and used these to compute values for health states.

**Results:**

Our total sample comprised 1,972 patients. Completion time was < 2 min for both methods. Both methods were scored as easy to perform. All DD coefficients were highly significant from the reference level (P < 0.001). For BW, however, only the second-level coefficient of “Cognition” was significantly different (*P* = 0.026). All DD coefficients were more precise with narrower confidence intervals than those of the BW method.

**Conclusions:**

Both the BW and DD are novel methods that are easy to apply. The DD method outperformed the BW method in terms of the precision of produced coefficients. Due to its task, it is free from a specific distorting factor that was observed for the BW method.

**Supplementary Information:**

The online version contains supplementary material available at 10.1186/s12874-023-02119-9.

## Introduction

Advances in medical treatments are improving survival and reducing key morbidities. Assessments of health status or health-related quality of life (HRQoL) are therefore becoming increasingly pertinent [1, 2]. Regulatory bodies including the US Food and Drug Administration (FDA) [3] and the UK National Institute for Health and Care Excellence (NICE) [4] actively encourage patient-reported HRQoL measurements alongside traditional clinical assessments.

Commonly patient-reported outcomes are used to assess HRQoL. As defined by the FDA, a patient-reported outcome is “any report of the status of a patient’s (or person’s) health condition, health behavior, or experience with healthcare that comes directly from the patient, without interpretation of the patient’s response by a clinician or anyone else.” The tools for such assessments are known as patient-reported outcome measures (PROMs) [5].

Instead of being provided by patients, however, the items currently included in most PROMs are based largely on expert opinion [6]. Moreover, many PROMs are based on classical test theory, using a basic measurement framework based on Likert scaling. Examples include the SF-36 [7], NHP [8], and the EORTC-QLQ-C30 [9]. These PROMs encompass multiple health domains, each scored by summing small sets of Likert items. Although such profiles provide useful information for many applications, they measure only the frequency or severity of the complaints of patients on separate health domains. To measure the impact of such complaints or limitations on perceived health status requires another type of measurement framework, often described as “preference-based”.

Preference-based PROMs explicitly incorporate weights reflecting the relative importance attached to categories or levels of specific health items (*item* is used here to reflect a specific aspect or feature of health; it can be synonymous with *attribute*, *domain*, or *dimension*). These weights can be combined to produce a single index that expresses the (social) value of a health state [10]. Such values can be meaningful in many research situations, such as monitoring patients’ health, assessing healthcare interventions, conducting cost-effectiveness analysis, and comparing health status across different populations [1, 11–13].

One drawback to the preference-based measurement framework is that it requires respondents to perform specific preference tasks entailing elements of assessment and judgment that are more complicated than those involved in conventional PROMs. As part of the assessment, respondents must make trade-offs in their evaluation of the health items. To ensure that respondents make such trade-offs properly, preference-based measurement also typically involves evaluating all these items together, and not item by item. In the next step, respondents must express a preference (judgment) in favor of one item (or set of items: i.e., health state) over the other. Complex, cognitively demanding preference tasks are likely to produce less accurate results. Such tasks should therefore be as simple as possible, nevertheless provide effective information [14].

Most existing preference-based measures in the social sciences and health sciences (psychology, economics, marketing, clinimetrics) are based on one of two fundamental measurement models for subjective phenomena: item response theory models and probabilistic choice models. It should be noted that these measurement models for subjective phenomena use an indirect form of measurement based on an underlying theory with a component of transforming the raw data. Therefore, such measurement models might be defined as an “internally consistent plan for developing a measure” [15]. These models are used to quantify such phenomena as attitudes, perceived health, intelligence, and consumer preferences. In these models the way preferences are elicited is crucial and can be done in different ways.

A new preference-based measurement model has recently been introduced: the multi-attribute preference response (MAPR) model [16–18]. It combines the Rasch model (item response theory) and the discrete-choice model (probabilistic choice). Most conventional preference-based methods applied in healthcare settings are based on hypothetical health states (often pairs) assessed by a sample of the general population. In contrast, the MAPR model allows patients to assess themselves, with the additional benefit of simplifying the vital preference tasks and making it possible to perform them on a smartphone. Although PROMs based on this methodology may appear simple to users, they involve several complex procedures, which we describe later in this article.

Drawing on the MAPR model, we initially developed the Better-Worse (BW) preference method. In light of findings from a previous empirical study [19], however, we decided to improve on this method and developed the Drop-Down (DD) method. In the current study, we explored whether respondents find the DD method easy to understand and complete, compared the interpretability and statistics of the estimated coefficients and the distribution of the computed health-state values of both methods.

## Methods

### Sample

We conducted a population-based, cross-sectional online survey. Respondents were patients (≥ 18 years of age) with one or more of in total 14 health conditions in the US involving pain, fatigue/sleep problems, mental health problems, respiratory diseases, diabetes, hearing or vision loss, eczema, gastrointestinal disease, heart disease, cancer, rheumatism, stroke, epilepsy, and other diseases. All had registered with Dynata, a market research company based in Rotterdam, the Netherlands. The sample was nationally representative for age, gender, and region. Those who completed the survey received a small financial compensation from Dynata, based on agreements between the company and the various respondent groups. Data collection took place from November to December 2020. Dynata provided demographic information and health conditions of the respondents.

### Health-outcome measure

**Fig. 1:**
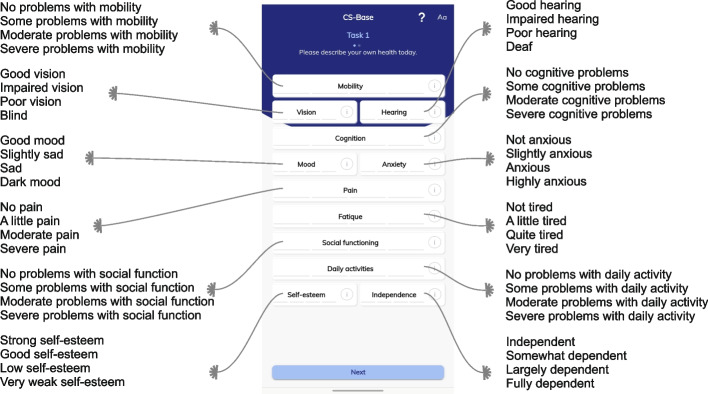
The 12 items of the generic health-outcome instrument CS-Base, each with four levels, as depicted in the HealthSnApp (an application for mobile phones)

We used the Château-Santé Base (CS-Base), a generic health-outcome instrument [20]. Developed for measuring health status or HRQoL, this instrument comprises 12 health items: mobility, vision, hearing, cognition, mood, anxiety, pain, fatigue, social functioning, daily activities, self-esteem, and independence (Fig. 1). Each item is specified at four levels (1,2,3,4). The CS-Base instrument is the first preference-based PROM that fully incorporated patient input in selecting the health items**.** In the study aimed at its development, 2256 patients with a wide range of health conditions were asked to select the most important items from a list of 47 candidate items, which were selected from existing generic measures [20]. The candidate items were presented to patients in an interactive and graphical diagram (HealthFan) reflecting different domains (e.g., social, mental, physical) and subdomains (e.g., discomfort, function, senses). The 12 most important items according to the patients were included in the CS-Base.

### Study design

This study comprised two arms, each entailing the use of the two preference methods (BW and DD, see below), albeit in reverse order. Dynata sent the two arms of our study (two links generated from our software HealthSnApp) to patients through their system, based on the Least Fill logic. In the “Least Fill” allocation logic, the system ensured that the links were distributed as evenly as possible among respondents.

### Mobile app

The full operation of the CS-Base is an electronic patient-reported outcome measure (ePROM) that uses special software and runs in the mobile application HealthSnApp (www.chateau-sante.info). It is operated by a cloud-based data collection technology that is new within the PROM field. The HealthSnApp (®, Patent) is a flexible tool, with interactive software routines and the potential of on-the-fly analytics. It runs on smartphones and computers, and it is highly configurable from a web-based console module. As part of our measurement model (MAPR), users perform two distinct tasks in the mobile app. The first produces a description (health state) of the patient’s current health condition (health status), and the second elicits the patient’s preference responses. For each preference method respondents performed both tasks.

### MAPR measurement model: response tasks

#### Task 1: description

**Fig. 2:**
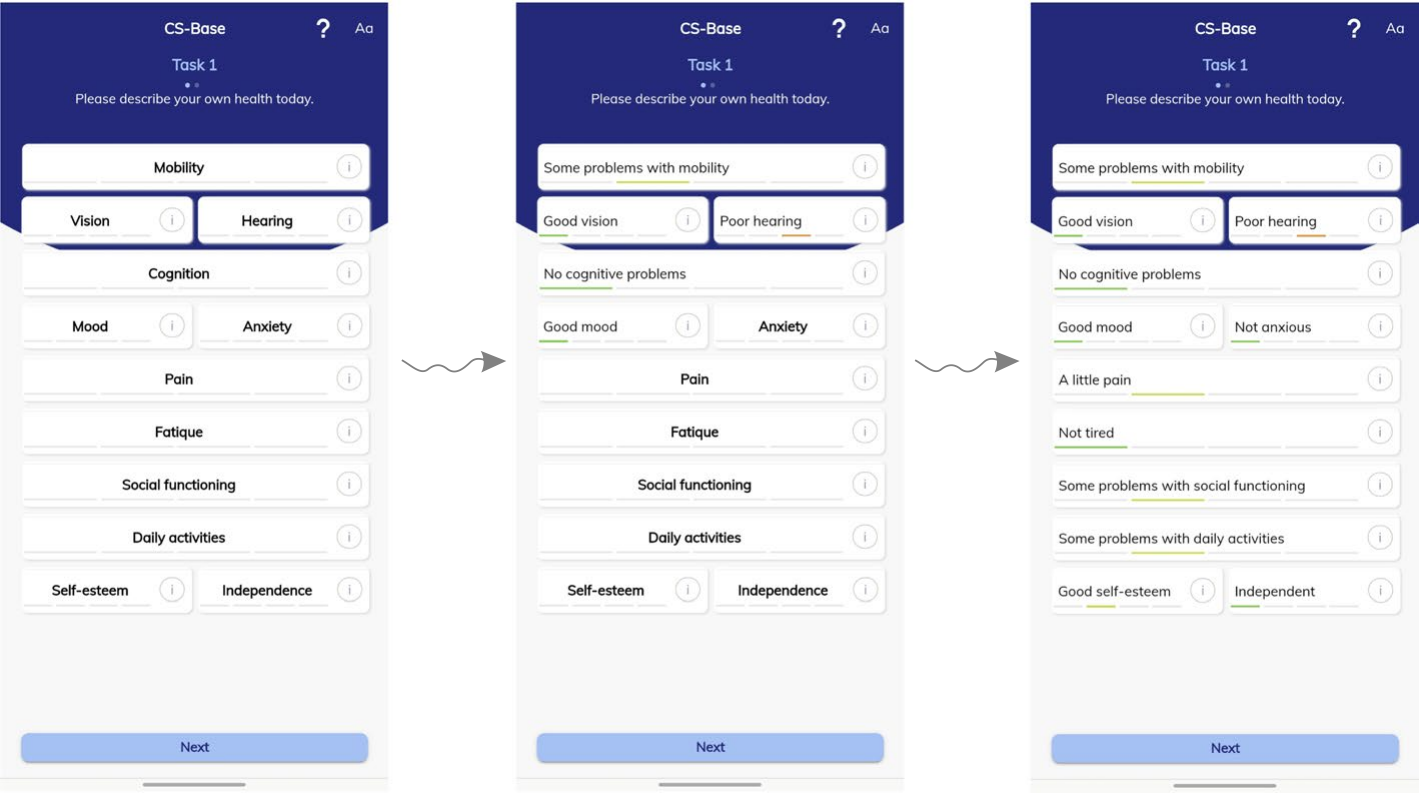
Screenshots of the CS-Base from the HealthSnApp during Task 1. In this descriptive task, all health items were listed in interactive (rotating) boxes presented on a single screen. When a patient selected the interactive box for a specific item, the box displayed response options. For example, when a patient selected the “Fatigue” box, the display shifted to offer the following response options: “Not tired,” “A little tired,” “Quite tired,” and “Very tired”

Each health item in the CS-Base is depicted in the app as an interactive box. All items are depicted together on one screen. When the patient clicks on the box for a specific health item, it rotates, displaying the response options (See demo: www.chateau-sante.info). For example, when a patient selected the “Fatigue” box, the display shifted (rotating) to offer the response option “Not tired” (Level 1). After selecting the box again, the display shifted to “A little tired” (Level 2), next to “Quite tired” (Level 3), and “Very tired” (Level 4). Patients rate their current health status by rotating the boxes to show the best-fit descriptions in all boxes (Fig. 2). The CS-Base app thus generates a description of a patient’s overall state of health expressed as 12 digits (e.g., 213111212221, or even worse 214111212331). Patients can also click information points beside the health items to access explanations.

#### Task 2: preferences

After Task 1, patients performed a second task (Task 2), based on their descriptions of their own health states (Task 1) according to the same CS-Base. Task 2 required them to make trade-offs and to provide preferences. In this study, we used two different preference methods in Task 2: Better-Worse (BW) and Drop-Down (DD).

### MAPR measurement model: preference methods

#### Better-Worse (BW) method

**Fig. 3:**
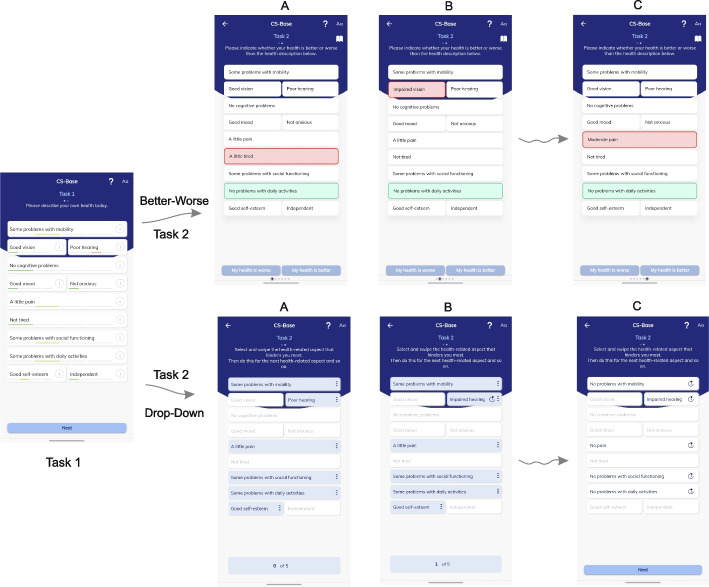
Screenshots of Task 2 from the Better-Worse (BW) and Drop-Down (DD) assessment and judgment tasks. For the BW method, respondents (i.e., patients) compared their own health states to five slightly different, alternative health states. With the exception of only two items, the alternative health states portrayed in Task 2 of the BW method did not differ from the actual health states as reported by the patients in Task 1. One of these items depicted an improvement of one level relative to the patient’s actual health state (depicted as a green box). Another item showed a reduction of one level relative to the patient’s actual health state (depicted as a red box). For the DD method, patients made multiple selections (2–5 times) of items at the levels that hindered or disturbed them the most. They did this by swiping (dropping down) the level and moving the item one level lower (i.e., better)

For the BW method, patients compared their own health states (Task 1) to a computer generated, slightly different alternative health state (Fig. 3). The alternative states differed from their own health states by a predetermined and limited number of items (in this study, two) that had been altered. Patients could thus regard these alternative states as hypothetical states. One of the items represented an improvement of one level compared to the patient’s actual health state (one level lower, depicted as a green box). The other item represented a reduction of one level compared to the patient’s actual health state (one level higher, depicted as a red box). For example, on Task 1, a patient reported being “Not tired” for the “Fatigue” condition and having “Some problems with daily activities” for the “Daily activities” condition. These two health items were altered into “A little tired” and “No problems with daily activities” to construct an alternative health state in the subsequent Task 2 (See Fig. 3A, Better-Worse). The generation of these alternative health states were based on a flexible randomization algorithm (number of alternative states (5 in this study), number of items to vary (2 in this study with distinct colors)) that was built into the console of the software that is used to steer the application (HealthSnApp).

The assumption is that a one-level improvement on one item is not necessarily the same difference as a one-level decline on another item. Patients may have appraised differences between the levels of distinct items in different ways. The patients were asked “Please indicate whether your health is better or worse than the health description below.” The task essentially called for the patients to internally make a trade-off between their own health state and other alternative states in a paired comparison, and then to select either their own health state or the alternative health state as better (i.e., 1 if preferred and 0 if not).

In this study patients compared five alternative health states with their own health state. For each patient, therefore, we had five sets of the most basic ranking, namely of two health states: 1,2 (coded as 1,0 for the statistical software; Additional file 1). The number of paired comparisons (BW) and drop-downs (DD: see below) was based on practical considerations, and particularly to reduce respondent burden.

#### Drop-Down (DD) method

For the DD method (Fig. 3), the patient’s own health state from Task 1 was presented on the screen. The patient was asked to select the item (with a suboptimal level: 2, 3, or 4) that hindered or disturbed them the most. By clicking or swiping (drop-down) this item is shifting one level lower (better). Each drop-down produced a health state ranked better than the initial health state from Task 1 (There had to be at least two items with levels > 1, otherwise the choice was predetermined. Items at Level 3 or higher could be dropped down more than once). Generating hypothetical health states is not needed in the DD method, as the DD task is based on patients’ own health states only.

In the DD method, patients make trade-offs between the levels of multiple items (i.e., is Level *i* of an item *x* worse than any level of another item?). In contrast to the BW method, patients do not have to make trade-offs between their own and an alternative health state.

Patients used the drop-down option up to a maximum of five times, with each drop-down producing a different (better) ranked health state. For each patient, therefore, the ranking for the states could range from 1,2,3 (with at least 2 items having suboptimal levels: 2 drop-downs) and 1,2,3,4,5,6 (in case of 5 drop-downs). The lowest or worst ranking was coded as 1, representing the patient’s actual health state.

In conventional valuation studies based on standard gambles, time trade-offs, or discrete choice experiments, a representative sample of the general population evaluates pre-determined health states using experimental designs to estimate all coefficients. However, the BW and DD methods do not depend on pre-defined health states. They involve patients with various health conditions, allowing coefficients to be estimated across the entire health status spectrum. If only a few patients report the worst level for a particular item, the coefficient can still be estimated, albeit with a wider confidence interval. If no patient reports a specific level for a particular item, it becomes impossible to estimate the coefficient.

### MAPR measurement model: statistical models

Like all probabilistic measurement models, the MAPR measurement model use an indirect form of measurement. The data generated by the preference methods are not measures. Ordinal response data (ranks) obtained from the preference methods are aggregated to estimate coefficients based on a mathematical (measurement) model. Subsequently, the coefficients are used to compute values for the health states. These values are the measures. The mathematical model consists of a latent (hidden) variable (the metric scale) and a set of manifests (observable) variables (i.e., the items of the CS-Base). Such models have a long history, commencing with the model developed by Louis Thurstone in 1927 [21]. Other researchers have introduced extensions to the basic Thurstonian model [22–25].

For all probabilistic measurement models, respondents must perform assessments (preliminary phase of information processing prior to making a judgment) and judgments (choice in favor of something) in particular ways to endorse specific responses. This then generates data for an analysis in accordance with the measurement model. Within these probabilistic measurement frameworks, the assessment consists of comparing at least two objects (i.e., health states or set of health items), with the aim of expressing which object is preferred (i.e., better). Therefore, the BW and DD methods are developed in such a way that both will produce preference data that fits the measurement model described below.

The data generated when patients select one health state over another (BW) are discrete-choice data. The data generated when patients rank health states from most favorite to least favorite (DD) are rank-ordered data. To process the data generated by the BW and DD methods, two different but related statistical models are adequate: conditional logit and rank-ordered logit. These models differ in terms of the expected data structure of health-state preferences and estimation procedures. The distinction is that the dependent variable (preference: choice or rank) in the conditional logit records only the best state by a value not equal to zero (in our case, we used the simplest variant, selecting the better of only two states). In contrast, the ranked-ordered logit model marks the rankings of the states.

#### Mathematics

The preference data of the BW and DD methods are processed in essentially the same way. The value of a health state *j* for an individual *i* is denoted by *V*_*ij*_. A respondent will rank state *j* higher than state *k* if *V*_*ij*_ > *V*_*ik*_. The probability of choosing state *j* as the most preferred of the set of *J* states (BW: 2; DD: 3–6) can be written as follows:

1 $${P}_{ij}= \frac{{e}^{{V}_{ij}}}{{\sum }_{k=1}^{J}{e}^{{V}_{ik}}}$$

The probability of observing a specific ranking among three or more health states (DD) can be written as the product of such terms, representing a sequential interpretation. In this sequence, the respondent first chooses the most preferred health state, followed by the most preferred of the remaining health states, and so forth. We assume that *V*_*ij*_ is a linear combination of the levels of the health-state items plus an error term *ε*_*ij*_ for the individual. The model is specified as follows:

2 $${V}_{ij}={\beta {\varvec{x}}}_{j}+{\varepsilon }_{ij}$$

where *β* represents a vector of regression coefficients. Further, ***x***_*j*_ is a vector of binary dummy explanatory variables (*x*^*δλ*^), with *δ* indicating one of the 12 items, *λ* indicating the levels of each of the items for a given health state. For example, in our study involving the CS-Base, *x*^*72*^ represents the second level (“A little pain”) of the seventh item (Pain). Because a given health state has the same expected value across all respondents, ***x*** is indexed only by *j*. Although the estimation procedures for the two models differ, they will produce the same results if the rank-ordered logit model is used for data consisting exclusively of sets of two states.

### Evaluation app and methods

After completing Task 2, patients answered five questions assessing their perceptions of ease-of-use for the app (HealthSnApp) and the relative difficulty associated with each of the two methods. The five questions concerned: (1) the clarity of the instructions provided in the app, (2) the overall experience of using the app, (3) the level of difficulty of the BW method, (4) the level of difficulty of the DD method, and (5) which method (BW or DD) patients find easier? Except for the last, binary question, scores for all other questions were 0–100 (with 0 indicating *not difficult at all* and 100 indicating *most difficult*).

### Analysis

#### Measurement model

In our study, the first level of each of the 12 CS-Base items (Level 1: no problems, or an optimal condition) was the reference level. We estimated regression coefficients for the remaining three levels (2, 3, and 4) using 36 dummy variables (12 × 3). We did not include any constants. Using a conditional-logit (McFadden’s) choice model (cmclogit, Stata 17.0), we estimated coefficients for the data derived from the BW method. For the DD data, we applied a rank-ordered logit choice model (cmrologit, Stata 17.0). We used the regression coefficients (weights) to compute the values for CS-Base health states. To allow for consistent comparison, the original values derived from the BW and DD methods were rescaled to 0.0 − 1.0, where 0.0 stands for the lowest value in their original scales (worst health state 444444444444) and 1.0 stands for the highest value (full health state 111111111111). Details for the values computation and rescaling are explained in Additional file 2.

#### Postulated states

**Fig. 4:**
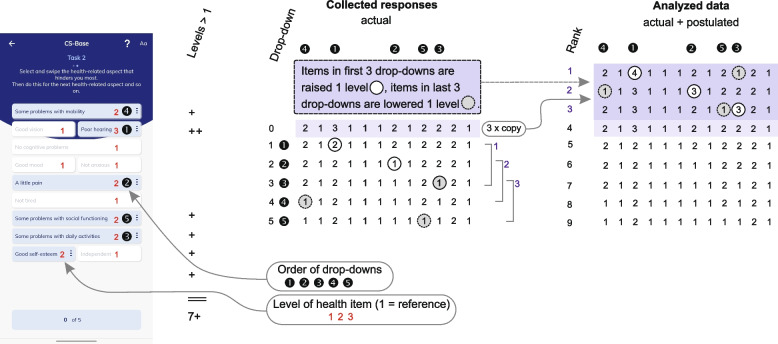
Schemes representing the steps taken to generate data for the Drop-Down analysis. The example reflects rated levels for the 12 items (with 7 levels being higher than the reference Level 1) of the CS-Base ePROM (left). Responses were stored on the server after five drop-downs (middle). The final data for the analysis were obtained after inserting postulated health states (right)

For the DD method, we created additional health states according to the patients’ responses. This step was necessary, as all the ranked health states produced in the preference task (in which patients dropped down item levels to improve their actual health states) were better than the actual health states of the patients. The information derived from this task was nevertheless limited. For example, although the case of five drop-downs (Fig. 4) generated six ranked health states, all the levels for these states were lower (i.e., better) than the patient’s actual health state. Moreover, for each of these ranked states, only one of the 12 items had a level that was lowered. Standard regression analysis requires variation (i.e., more than one item must vary for each health state, and to both lower and higher levels) to determine a stable estimation (i.e., achieve convergence). For this reason, we imputed postulated states based on analytical information derived from the actual states (Fig. 4). This extended the ranking in the analyses to a maximum of 9 ranked states.

#### Evaluation questions

We calculated mean scores for the evaluation questions. For the completion time of the BW and DD methods, as recorded on the server, we tested differences according to paired t-tests. To explore the relationship between age and the completion time of the BW and DD methods, we computed Pearson correlations. We also explored the difference between age groups in terms of educational level by using Fisher’s exact test. To explore the impact of sociodemographic factors in evaluating the level of difficulty of the two methods, we used a t-test (gender) and ANOVA tests (age groups, education). We tested the relationship between age and the level of difficulty of the two methods according to Pearson correlations. For the binary question (“Which method did you find easier?”), we used the Fisher exact test. We also used t-tests and the Fisher exact test for order effects. The software packages that we used to compute and visualize our results were SPSS 25.0, Stata 17.0, and CorelDraw 22.0.

## Results

### Sample

In all, 1,942 patients participated in our study. Of these patients, 15 were excluded because they had participated in only one of the two methods. The final sample comprised 1,927 patients, all of whom had completed both the BW and DD methods. In the analysis for deriving coefficients, however, we excluded 538 (371 with BW, 412 with DD, 245 overlapped in both methods) patients who rated their health status as full health (i.e., 111,111,111,111, Level 1 for all 12 items) in Task 1 (respondents proceeded to Task 2 only if their reported health in Task 1 was suboptimal). This left 1,389 patients.

### General information of patients

The mean and median ages of the patients were both 46 years, with ages ranging from 18 to 89 years. There were 1,023 (53.3%) female patients. More than half of the patients had more than secondary education (high education level) in our study (Table 1). Elderly (≥ 58 years) were higher educated (more than secondary education) than younger patients (18–57 years), namely 78% versus 40% (*P* < 0.001, Additional file 3). All 1927 patients reported one or more of 14 health conditions (Table 1). The majority (893, 46%) reported pain, followed by fatigue/sleep problems (763, 40%), mental health problems (451, 23%), respiratory diseases (418, 22%), and diabetes (364, 19%). A check revealed that the 1,389 patients included in the statistical analysis to estimate the coefficients were quite similar to the total sample of 1,927 patients in terms of their socio-demographics (Additional file 4).

**Table 1:** Characteristics of the total sample and of the separate study arms (I and II)

Characteristics	Study arm I (970)	Study arm II (957)	Total sample (1,927)
Gender, N (%)	964 (99)	956 (100)	1,920 (100)
Female	523 (54)	500 (52)	1,023 (53)
Male	441 (46)	456 (48)	897 (47)
Age (year), N (%)	964 (99)	957 (100)	1,921 (100)
18–27	182 (19)	161 (17)	343 (18)
28–37	172 (18)	189 (20)	361 (19)
38–47	151 (16)	164 (17)	315 (16)
48–57	135 (14)	168 (18)	303 (16)
58–67	174 (18)	157 (16)	331 (17)
68–77	119 (12)	99 (10)	218 (11)
≥ 78	31 (3)	19 (2)	50 (3)
Ethnicity, N (%)	970 (100)	913 (95)	1,883 (98)
Asian/Asian-American	38 (4)	47 (5)	85 (5)
Black/African-American	112 (12)	80 (9)	192 (10)
Hispanic or Latino American	55 (6)	58 (6)	113 (6)
Native American/Inuit/Alaskan	18 (2)	13 (1)	31 (2)
Native Hawaiian/Pacific Islander	4 (0)	3 (0)	7 (0)
White American/Caucasian	731 (75)	698 (77)	1,429 (76)
Other	12 (1)	14 (2)	26 (1)
Education^a^, N (%)	609 (63)	623 (65)	1232 (64)
More than secondary school	490 (51)	505 (53)	995 (52)
Secondary school graduate	102 (11)	102 (11)	204 (11)
Less than secondary school	17 (2)	16 (2)	33 (2)
Health conditions^b^, N (%)	970 (100)	957 (100)	1,927 (100)
Pain	432 (45)	461 (48)	893 (46)
Fatigue/sleep problems	354 (37)	409 (43)	763 (40)
Mental health problems	225 (23)	226 (24)	451 (23)
Respiratory disease	211 (22)	207 (22)	418 (22)
Diabetes	194 (20)	170 (18)	364 (19)
Hearing or vision loss	148 (15)	164 (17)	312 (16)
Eczema	115 (12)	125 (13)	240 (13)
Gastrointestinal disease	74 (8)	102 (11)	176 (9)
Heart disease	58 (6)	67 (7)	125 (7)
Cancer	71 (7)	48 (5)	119 (6)
Rheumatism	42 (4)	42 (4)	84 (4)
Stroke	28 (3)	26 (3)	54 (3)
Epilepsy	23 (2)	24 (3)	47 (2)
Other diseases	113 (12)	129 (14)	242 (13)

^a^Educational backgrounds were missing for about 36% of the respondents, as Dynata had incomplete data. Only a few respondents did not provide information on age (1%) or ethnicity (5%)

^b^Respondents can have multiple health conditions

### Evaluation app and methods

The mean (SD) scores of the total sample for the four rating questions were as follows: Clarity of instructions 33 (28); Overall experience of use 28 (26); Level of difficulty – BW method 30 (26), – DD method 29 (26). In response to the question, “Which method [BW or DD] did you find easier?”, 1,036 patients (54%) selected BW as the easier method. No order effects between the two study arms were observed for the evaluation questions (Additional file 5). The mean completion time for the BW method was 80 s and was 99 s for the DD method, they were significant different (*P* = 0.008; Additional file 6).

We performed separate analyses for three questions (level of difficulty – BW/DD method; which method did you find easier?) to explore possible associations between the sociodemographic characteristics of patients and various evaluations. No significant differences were found between subgroups of gender and education (Additional file 7). However, statistically significant difference was found between age groups divided by 58-year. The mean scores of younger (18–57 year) and older (≥ 58 year) age groups were 31 and 28 (*P* = 0.008) on the difficulty of BW method, 30 and 26 (*P* = 0.001) on the DD method, their proportions of selecting BW as easier were 56% and 51% (*P* = 0.042). Very weak decreasing linear relationship were found between age and the level of difficulty of the two methods (BW: *r* = 0.028, *P* = 0.227; DD: *r* = -0.057, *P* = 0.013; Additional file 8), as well as between age and completion time (BW: *r* = 0.073, *P* = 0.007; DD: *r* = 0.057, *P* = 0.036; Additional file 9).

### Health states

The total number of possible health states that can be generated by the CS-Base is 16,777,216 (4^12^, 12 items each with 4 levels). By Task 1 in total, 1,184 different health states were reported in the BW method and 1,123 health states in the DD method. There were 678 patients who rated identical health states by both methods.

The full health state (111111111111) was reported by 371 (19.3%) patients in the BW method and 412 (21.4%) patients in the DD method. The state (444444444444) was reported by two patients in the DD method, but not by any patient in the BW method. The most frequently reported health state (besides the full health state) in both methods was 111111211111: for 57 patients (3.0%) in BW and for 55 patients (2.9%) in DD. The worst health state in the BW method was 443414444444 (1 patient) and 444444444444 in the DD method (2 patients). Mild impaired health states (with only 1 or 2 of the 12 items at Levels 2 or 3) were most common reported in both methods.

### Statistical model and coefficients

For the BW method, the total number of paired comparisons (Task 2) for the 1,389 patients was 6,945 (five paired comparisons for each patient in Task 2). Patients selected their own health state as the better state in the comparisons on 4,684 occasions (67.5%). For the DD method, the total number of ranked health states (Task 2) for the 1,389 patients was 5,709.

**Fig. 5:**
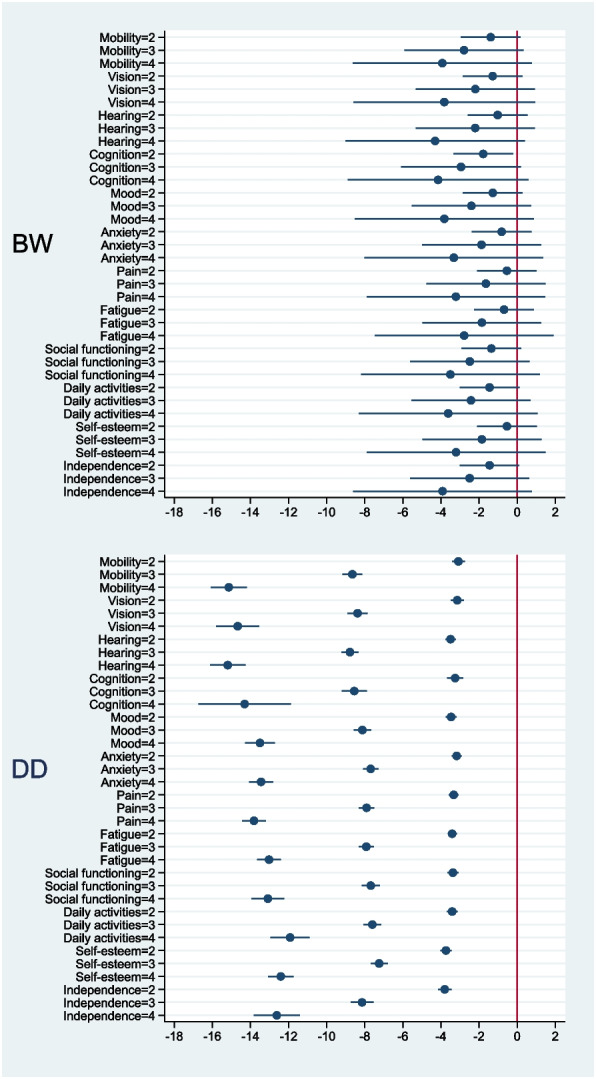
Distribution of coefficients and their 95% confidence intervals for the Better-Worse (BW) and Drop-Down (DD) methods

The BW method needed three iterations in the statistical analysis to reach convergence, while the DD method needed eight. For both methods, all the coefficients revealed a logical order (slight, moderate, and severe problems). None of the BW coefficients indicated statistically significant differences from the reference level (Table 2), apart from Level 2 of “Cognition” (*P* = 0.026). In contrast, all DD coefficients indicated statistically significant differences (*P* < 0.001). Standard errors of the coefficients for the BW method were relatively large compared with those for the DD method. For the DD method, standard errors varied over the items and their respective levels. For the BW, standard errors of each of the distinct levels were almost equal on all items. Moreover, differences between the levels were almost equal on all items. The DD method revealed distinct and uniform differences in coefficients between levels for all items (Fig. 5). The confidence intervals for the DD coefficients were smaller than those for the BW coefficients. Only Level 4 of “Cognition” showed a larger confidence interval, which seems due to the low number of responses collected at that level, in combination with a low number of generated postulated health states (Additional file 10).

**Table 2:** Coefficients derived from the Better-Worse and Drop-Down methods (*N* = 1389)

Item levels	Better-Worse			Drop-Down		
	Coefficient	SE	P	Coefficient	SE	P
Mobility (2)	˗1.39	0.80	0.081	˗3.09	0.17	< 0.001
Mobility (3)	˗2.79	1.60	0.080	˗8.65	0.26	< 0.001
Mobility (4)	˗3.93	2.40	0.102	˗15.14	0.48	< 0.001
Vision (2)	˗1.29	0.80	0.106	˗3.15	0.18	< 0.001
Vision (3)	˗2.20	1.60	0.169	˗8.38	0.27	< 0.001
Vision (4)	˗3.83	2.40	0.116	˗14.67	0.58	< 0.001
Hearing (2)	˗1.02	0.80	0.199	˗3.49	0.14	< 0.001
Hearing (3)	˗2.20	1.60	0.167	˗8.78	0.23	< 0.001
Hearing (4)	˗4.31	2.40	0.073	˗15.20	0.47	< 0.001
Cognition (2)	˗1.78	0.80	0.026	˗3.25	0.22	< 0.001
Cognition (3)	˗2.95	1.60	0.066	˗8.55	0.34	< 0.001
Cognition (4)	˗4.15	2.42	0.087	˗14.31	1.23	< 0.001
Mood (2)	˗1.28	0.80	0.109	˗3.47	0.14	< 0.001
Mood (3)	˗2.40	1.60	0.132	˗8.12	0.23	< 0.001
Mood (4)	˗3.82	2.40	0.110	˗13.50	0.40	< 0.001
Anxiety (2)	˗0.82	0.80	0.307	˗3.17	0.13	< 0.001
Anxiety (3)	˗1.87	1.59	0.241	˗7.69	0.21	< 0.001
Anxiety (4)	˗3.32	2.40	0.165	˗13.44	0.32	< 0.001
Pain (2)	˗0.54	0.80	0.498	˗3.33	0.13	< 0.001
Pain (3)	˗1.64	1.59	0.302	˗7.91	0.21	< 0.001
Pain (4)	˗3.22	2.39	0.178	˗13.82	0.32	< 0.001
Fatigue (2)	˗0.69	0.80	0.388	˗3.41	0.12	< 0.001
Fatigue (3)	˗1.85	1.59	0.245	˗7.92	0.20	< 0.001
Fatigue (4)	˗2.78	2.39	0.244	˗13.03	0.32	< 0.001
Social function (2)	˗1.36	0.80	0.089	˗3.37	0.15	< 0.001
Social function (3)	˗2.48	1.60	0.119	˗7.68	0.24	< 0.001
Social function (4)	-3.50	2.39	0.143	˗13.09	0.44	< 0.001
Daily activities (2)	˗1.45	0.80	0.069	˗3.41	0.14	< 0.001
Daily activities (3)	˗2.42	1.60	0.128	˗7.60	0.24	< 0.001
Daily activities (4)	˗3.62	2.40	0.131	˗11.92	0.52	< 0.001
Self-esteem (2)	˗0.54	0.80	0.502	˗3.74	0.15	< 0.001
Self-esteem (3)	˗1.85	1.60	0.246	˗7.24	0.23	< 0.001
Self-esteem (4)	˗3.21	2.39	0.180	˗12.41	0.34	< 0.001
Independence (2)	˗1.45	0.80	0.069	˗3.80	0.18	< 0.001
Independence (3)	˗2.49	1.60	0.118	˗8.14	0.30	< 0.001
Independence (4)	˗3.92	2.39	0.101	˗12.62	0.62	< 0.001

*SE* Standard error

We observed more sensible differences among the coefficients for the DD method than for the BW method. For example, all 12 coefficients for Level 3 of the DD method were lower than any of the 12 coefficients for Level 2. This was not the case for the BW method. For example, pain is known as a main determinant of health status, nevertheless the coefficient for Level 2 for “Cognition” was lower than Level 3 of “Pain”. For the BW method we also observed that Level 4 for “Pain” had a relatively low coefficient (− 3.22), whereas the Level 4 coefficients for “Social functioning” and “Daily activities” were even lower. Inspection of Table 2 (See also scatterplot: Additional file 11) showed a wider range of the BW coefficients for Level 2 and Level 3 in comparison with the DD.

### Health-state values

**Fig. 6:**
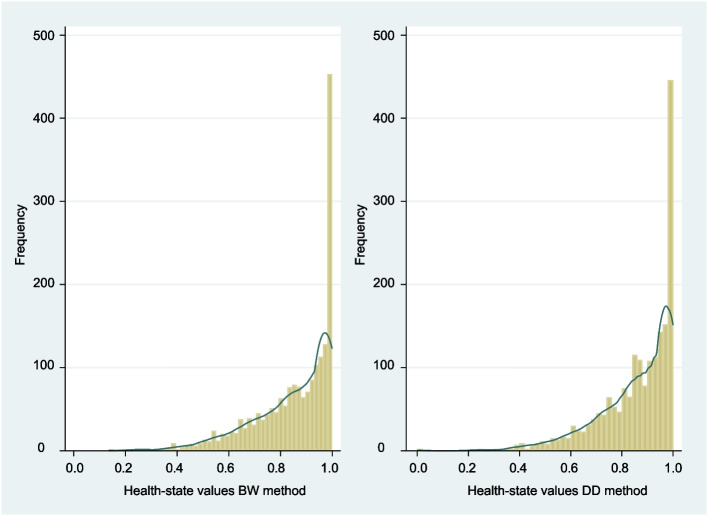
Distribution of computed values, based on the estimated regression coefficients, of all health states reported by the patients for the Better-Worse (BW: 1,184) and Drop-Down (DD: 1,123) methods

**Fig. 7:**
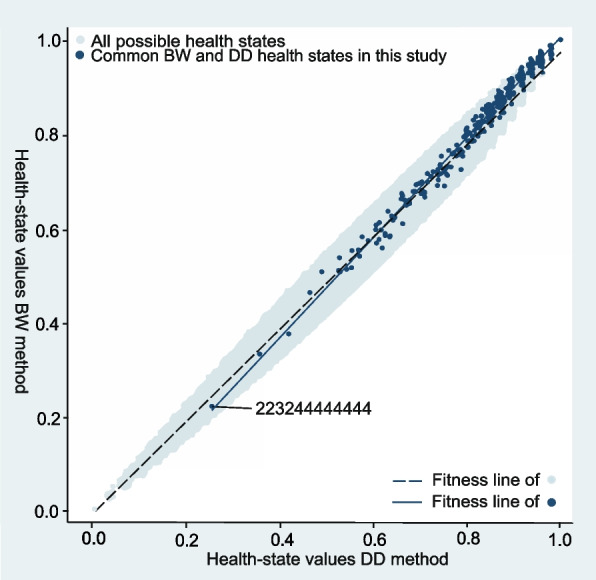
Computed values for health states in the CS-Base ePROM, based on the estimated regression coefficients from the Better-Worse (BW) and Drop-Down (DD) methods for all possible health states (16,777,216; depicted as light blue dots) and the common BW and DD states (328) reported by the patients in this study (dark blue dots)

Overall, the distribution of the computed health-state values for the two methods was similar (Fig. 6). Based on all possible CS-Base health states which ranges from the full state (111111111111) to the state 444444444444, the originally computed BW values ranged from − 43.60 (state 444444444444) to 0.0 (state 111111111111), the DD values ranged from − 163.12 to 0.0. The rescaled BW and DD values for all possible CS-Base states both ranged from 0.0 to 1.0. Based on the health states reported in this study, the rescaled BW values ranged from 0.14 (originally − 37.67, state 443414444444) to 1.0 (state 111111111111). The rescaled DD values ranged from 0.0 (state 444444444444) to 1.0. The correlation between the generated values for the two preference methods was high (Fig. 7). For the complete set of computed health states: *r* = 0.987; for the common BW and DD health states as reported by the patients: *r* = 0.993.

## Discussion

One major challenge in the field of healthcare involves the development of preference-based PROMs that are simple to use, while also producing consistent and credible results. In our investigation, we conducted a head-to-head comparison of two novel, preference-based methods (BW and DD) for measuring the health status of patients. According to the evaluation, patients evaluated both our app and the two methods overall as accessible and unproblematic in use. However, the statistical performance of the DD method and the interpretability of its results appeared to be better.

When using the BW method, patients were disproportionately likely to identify their own health states as the preferred (better) state [19]. We could logically expect patients to prefer their own health states in 50% of the cases. It appears that many of them are reluctant to any decline on a health item, even if this goes together with an improvement on another health item. Because of this distorting factor—and possibly other features of the method—the BW data were capturing less information. More specifically, patients apparently regard their own health states as better than the alternative, hypothetical states, due to risk aversion and uncertainty. In a series of experiments, Kahneman and Tversky (1979) demonstrated that individual losses weighed heavier than gains in decision-making [26]. One implication of this phenomenon (i.e., “loss aversion”) is that patients have a strong tendency to remain at a status quo, as the disadvantages of leaving it appear to outweigh the advantages [27]. This unwillingness of patients to make balanced trade-offs between their own health states and other health states was the main obstacle encountered in using the BW method.

The ultimate results after the different steps in our measurement model are the weights (coefficients) that are produced. Standard errors for the coefficients generated by the DD method were relatively small, and differences relative to the reference level were statistically significant for all coefficients. The BW method generated contrasting results, as the standard errors were relatively large and only one coefficient was statistically significant different from the reference level. The observed ‘status quo’ factor (it might be a bias, but for now we are not certain whether this factor is producing systematic differences) might be an important cause for the relatively large standard errors of the coefficients, even with a sample size of 1,389 patients. Another explanation might be that in this study for the BW method the alternative states in the preference tasks differed only on two of the 12 items (one item a level higher, another item a level lower). This may have introduced multicollinearity into the design matrix of the statistical analysis. The BW method needed only three iterations to reach convergence, which we consider, based on our experience from previous studies, suspicious. For the BW method we also observed an extraordinarily high coefficient on Level 4 for “Pain” that is not supported by literature. The DD estimation is based on the ranking of multiple states (in this study, two to five ranked states, plus additional postulated states up to a maximum of nine ranked states) as opposed to the paired-comparison data (five sessions, each having two ranked states) used in the BW method. The DD method thus produces more powerful information. We have initiated validation and empirical studies based on the DD method, employing the same outcome measure, CS-Base, as was used in this study [13, 28].

Although the coefficients of the two methods showed differences among the items, the computed health-state values were rather similar. It seems that despite the coefficients of the BW method are less precise, the relative difference across levels of all the 12 items are quite comparable between the BW and DD method. Therefore, coefficients for all 12 items together are producing health-state values that are quite similar.

In the BW method, patients compared their own health state to hypothetical states. In each of these hypothetical health states, there are two items different from the patient’s own health state: one item is lowered one level (milder problems), another is increased one level (severer problems). Such hypothetical health states might be difficult for patients to imagine. The situation was simpler for the DD method. In the DD method, patients only have to assess their own health state and to indicate which item they consider as most disturbing. The states presented to patients during the various drop-downs steps were states that they could probably imagine or might even have experienced. As such, the DD method can be seen as more experience based.

Although it does seem that the typical distorting factor (status quo) that we observed for the BW method is not present in the DD method, we cannot rule out the possibility that other distorting factors or biases may play a role in the task of the DD method. Because the DD task is so simple and so connected to the natural experience of patients (“what is disturbing me most in my disease state?”), however, we consider it as highly unlikely that a significant factor or bias mechanism would play a role in the background.

The DD method shares some similarities with a well-endorsed alternative to conditional ratings and ranking methods: the best–worst scaling (BWS) approach developed by Louviere and colleagues [29]. In the BWS method, individuals choose their most and least preferred options from a range of alternatives. In contrast, the DD method requires patients only to choose their worst item. In theory, patients might also be able to choose their best item. In our study, however, we felt that asking patients to choose the “best” (or least worst) health limitation would make the task complex and counter-intuitive. However, it is not possible to perform assessment tasks using BWS similar to the BW or DD methods. In BWS hypothetical states are presented that are generated based on an experimental design. Respondents in BWS studies are often not patients. In the DD or BW method, all respondents are patients. Patients are asked to describe their own health states followed by the preference-based tasks (BW, DD). In the BW method hypothetical states are presented to patients that are slightly different from their own health states. In the DD method, only patients' own health states are presented but no hypothetical states.

In addition to the measurement model used in our study, health economists use other specific valuation techniques for obtaining preference-based measures in healthcare. These valuation techniques generate utilities for health states. Utilities are a special type of “values”, as they are anchored on “full health” and “dead” at respectively 1.0 and 0.0. Utilities can be used for calculating quality-adjusted life years (QALYs) that are often used in economic evaluations. Values generated in this study are not suitable for computing QALYs as “dead” is not positioned on the scale. Such “utility” techniques (e.g., standard gamble and time trade-off) are inherently more complicated, as their goal is to produce an anchored scale (e.g., with a score of 0 representing “dead”) [30]. In addition to health items, the integration of various aspects (e.g., “dead” and/or “giving up life years”) into these utility techniques as additional attributes, substantially complicates the assessment. For many research questions, however, there is no need to include “dead” on the scale. For example, the scale for the CS-Base extends from the best to the worst CS-Base health state, thereby providing sufficient information for many applications. Compared to methods that are applied in health economics, the preference tasks we present here are much simpler. The health-state values calculated for CS-Base using the BW and DD methods are suitable for cost-effectiveness studies but cannot be directly applied in generic cost-utility analyses based on QALYs. To do so, additional methods are required to rescale the values generated by the BW or DD method into utilities [31].

Despite the usefulness of its findings, our study is subject to several important limitations. First, the patients participating in the study were recruited by a market research company. They might therefore have had insufficient motivation, which could have affected the robustness of our results. For example, we observed differences between the two identical assessments of their own health status (Task 1) as performed for BW and DD. Yet, in true clinical settings or medical health surveys, patients would probably be better motivated to pay careful attention to the instructions and tasks. Second, the education levels of our patients were not nationally representative, older patients in our study had higher education levels than younger patients. Our sample was representative regarding age and gender, but we did not deliberately seek representativeness regarding education. This might explain why older patients in our study regarded the two methods easier than younger patients. However, several studies have found that education has a limited impact on how individuals or populations assess and value different health states [32–34]. A third limitation to our study is that we did not consider interactions between the items. This might have reduced the accuracy of the estimated coefficients and values [35]. The CS-Base comprises 12 items, each with four levels. Although this results in a comprehensive generic PROM, it also generates a large number of interactions (two-way interactions alone numbered 630). The number of responses in our study, at least for the CS-Base, was insufficient to estimate all two-way interaction coefficients. In the future, larger studies or PROMs with fewer items/levels might allow the consideration of interactions between items. All tasks in our study were administrated through an online survey. The online administration mode using software has some advantages, such as enhancing accessibility as it eliminates geographical constraints. Respondents can conveniently participate from any location with internet access. It also mitigates time constraints, for instance, we could still conduct the study during the Covid pandemic period. However, online surveys might also have drawbacks compared to face-to-face interviewing, for example, since researchers have less supervision over the conditions under which respondents engage with the research tasks.

Interest and investments in the development of tools and methods relying on artificial intelligence (AI; self-learning) are increasing. We expect that AI will also have a place in the development and use of ePROMs. For example, in the essential algorithm that we used to create postulated health states for the DD method, the item levels were raised or lowered by assuming that the differences between levels were equal. It could nevertheless be possible to steer the procedure toward selecting the items to alter by performing a cycle of analyses using the information on the coefficients estimated during each cycle. The results could then serve as input for the next cycle. In this case, items could be raised or lowered by considering possible interval differences in the steps between item levels.

We have identified several advantages of the DD method over the BW method. For example, it clearly produces regression coefficients with relatively small standard errors and with distinct and uniform differences between the levels of the coefficients. It could therefore be appropriate for smaller studies. As revealed by a sub-analysis of the study sample, even 200 patients would have been sufficient to replicate the coefficients. Another advantage of the DD method is that it does not require a randomization procedure to generate the alternative health states used in Task 2 of the BW method (thereby reducing the need for network connection and data transmission). A further benefit is that the DD method does not require patients to remember how they previously described their own health status. Perhaps most importantly, the DD method is not susceptible to the disturbing response factor observed in the BW data. Although it does require somewhat more time (maybe because patients pay more attention to the task) to complete than the BW method does, it generally takes less than two minutes, and it is relatively easy to assess. Finally, the DD method offers relevant clinical information about the health aspects that individual patients regard as most disturbing. As for the BW method, although it did not perform as well as the DD method in our setting of health status measurement, it might be appropriate and useful for other settings (e.g., marketing) that are not susceptible to the status-quo factor. Moreover, small modifications to the DD and BW methods could give rise to useful new tools for use in a variety of settings.

## Conclusions

Our two new methods (BW and DD) are simpler to use than most conventional, preference-based methods. Patients, even older ones, evaluated both methods as relatively simple and easy to understand. The DD method nevertheless clearly outperforms the BW method in terms of the precision of produced coefficients. Researchers are planning further studies to gather additional evidence on the use of the DD method. Other plans call for further improvements to the assessment and judgment tasks in the DD, as well as refinements to the user interface to make the tasks more attractive and understandable, and simpler.

### Supplementary Information

**Additional file 1. **Scheme data structure BW DD.

**Additional file 2. **Values computation and rescaling.

**Additional file 3: Table A3. **Difference between age groups in terms of educational level.

**Additional file 4: Table A4. **Characteristics of the total sample and of the sample excluding patients who reported full health state.

**Additional file 5: Table A5. **Order effects (two study arms) on the difficulty of the BW and DD methods.

**Additional file 6: Table A6. **Completion time (seconds) for the Better-Worse (BW) and Drop-Down (DD) methods.

**Additional file 7: Table A7. **Impact of sociodemographic factors on the assessment of the difficulty of the BW and the DD methods.

**Additional file 8: Table A8. **Correlation between age and difficulty scores for the BW and DD methods.

**Additional file 9: Table A9. **Correlation between Age and completion time (second) for the BW and the DD methods (*N*=1384).

**Additional file 10: Table A10. **The number of observations of each level of CS-Base items by BW and DD methods (*N*=1927).

**Additional file 11: Figure A11. **Scatter plot of BW and DD coefficients.

## Data Availability

The anonymized dataset is available from the corresponding author.

## Abbreviations

BW: Better-Worse method
DD: Drop-Down method
MAPR: Multi-attribute preference response
ePROM: Electronic patient-reported outcome measure
HRQoL: Health-related quality of life
SE: Standard error
