# Supplementary material for: From simple to even simpler, but not too simple: a head-to-head comparison of the Better-Worse and Drop-Down methods for measuring patient health status
Source: BMC Med Res Methodol. 2023 Dec 16;23:299. doi: 10.1186/s12874-023-02119-9 (PMC10725035; doi:10.1186/s12874-023-02119-9)

Additional file 1

**Scheme**

Data structure generated in Task 2 from BW and DD method

In Task 1 of the HealthSnApp software application a respondent (i.e., patient #538) has described (here based on CS-Base PROM) the actual health status as a CS-Base health state “213111212221” (12 items: Item1 ... Item12). The data structure generated in Task 2 based on the Better-Worse (BW) and the Drop-Down (DD) methods is presented below.

For the BW method, respondents compare their own health states to a small set of slightly different, alternative health states. In this example, respondents performed five comparisons (for each alternative state, the respondents’ own health states are copied to create pairs [Pair], which are needed for the analysis). The alternative health states differed from the actual health states classified by the respondents (Task 1) for only two items. One of these items depicted an improvement of one level (bold) relative to the respondent’s actual health state (one level lower). The other item depicts a reduction of one level (underlined) relative to the respondent’s actual health state (one level higher). The generation of these alternative health states is based on a flexible randomization algorithm (number of alternative states, number of items to vary, colors) built into the software, with the variable Pref capturing the rankings (0,1=1,2). Ranking 1 is the preferred (better) health state.

For the DD method, respondents made five selections of items (variable Drop) at the levels that hindered or disturbed them the most. They did this by swiping (dropping down) the level and moving the item one level lower (i.e., better). The rankings are stored in the variable Pref: a ranking of 6 corresponds to the “best health state;” a value of 5 correspondents to “second-best;” and so forth. Ranking 1 is the respondent’s own health state from Task 1. See also Figure 4 in the article.


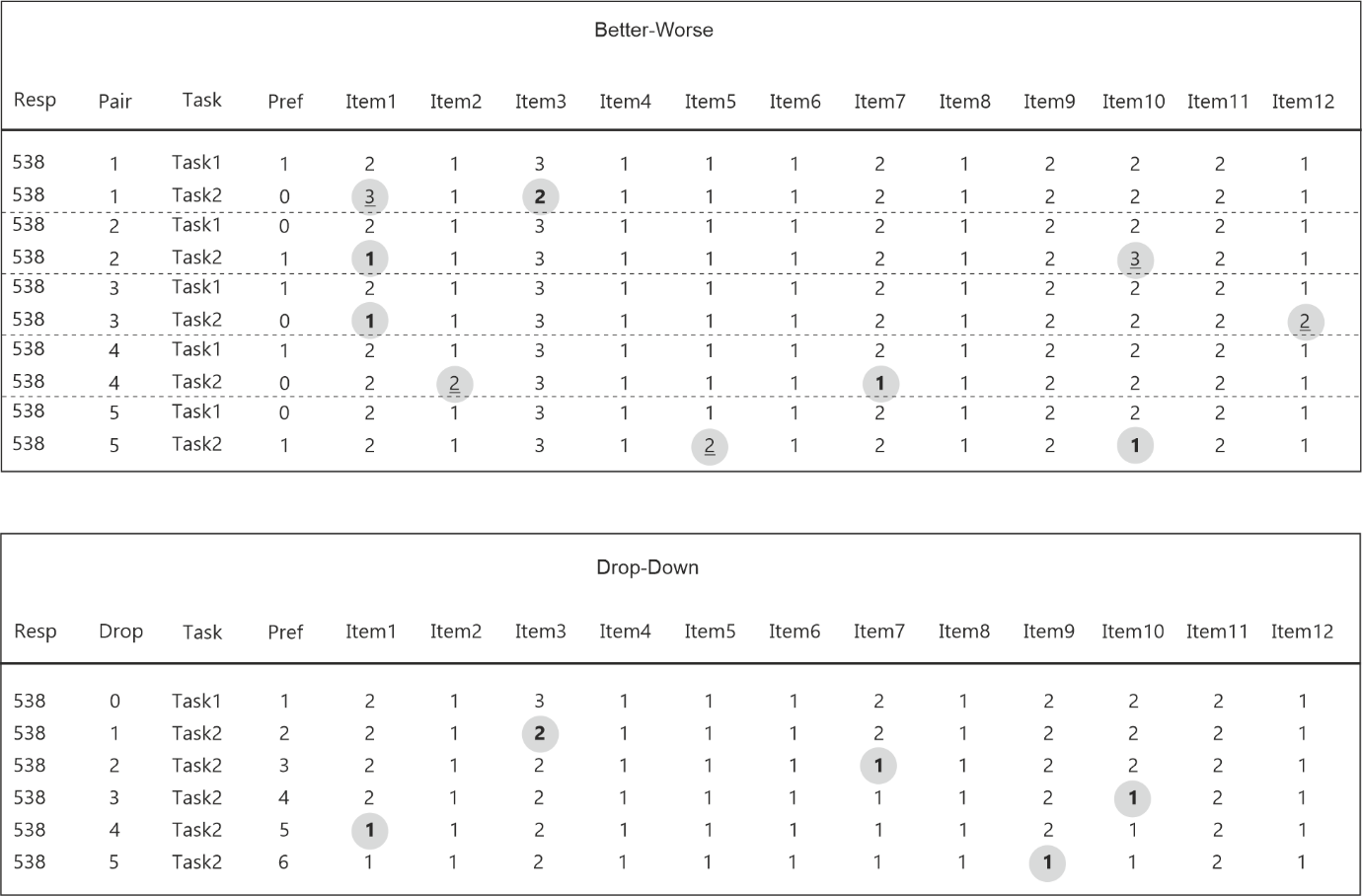

Supplement: Supplementary file 1 — Additional file 1. Scheme data structure BW DD. [file 12874_2023_2119_MOESM1_ESM.docx]
