# Supplementary material for: From simple to even simpler, but not too simple: a head-to-head comparison of the Better-Worse and Drop-Down methods for measuring patient health status
Source: BMC Med Res Methodol. 2023 Dec 16;23:299. doi: 10.1186/s12874-023-02119-9 (PMC10725035; doi:10.1186/s12874-023-02119-9)
Supplement: Supplementary file 3 — Additional file 3: Table A3. Difference between age groups in terms of educational level. [file 12874_2023_2119_MOESM3_ESM.docx]

Additional file 3

**Table A3**

Difference between age groups in terms of educational level

| **Education** | **Age groups divided at 58y**  **N (% within column)** | | **Age groups divided at 46y**  **N (% within column)** | |
| --- | --- | --- | --- | --- |
|  | **18–57y** | **≥ 58y** | **18-46y** | **≥ 46y** |
|  | P < 0.001* | | P < 0.001* | |
| More than secondary school | 527 (40) _a_ | 465 (78) _b_ | 371 (39) _a_ | 621 (64) _b_ |
| Secondary school graduate | 133 (10) _a_ | 68 (11) _a_ | 90 (10) _a_ | 111 (11) _a_ |
| Less than secondary school | 28 (2) _a_ | 5 (1) _b_ | 18 (2) _a_ | 15 (2) _a_ |
| Missing | 634 (48) _a_ | 61 (10) _b_ | 464 (49) _a_ | 231 (24) _b_ |

y = years

*Fisher’s exact test was used to determine whether a significant statistic difference existed between two age divisions for older and younger respondents (18–57y vs ≥ 58y; 18-46y vs ≥ 46y) regarding education levels of respondents.

- Any cell with a subscript letter (_a, b_) denotes a subset of age groups whose column proportions do not differ significantly from each other at the 0.05 level.
- Cells without the same subscript letter (_a, b_) denote subsets of age groups whose column proportions differ significantly from each other at the 0.05 level.
- The 58-year age break is based on the values of seven age groups. The values for the three older age groups (58–67 years, 68–77 years, ≥ 78 years) were clearly larger than those for the younger age groups.
- The 46-year age break is based on median age.

**Results report**

There was a statistically significant difference between the 18–57 y and ≥ 58 y groups (and between the 18–46 y and ≥ 46 y groups) (p < 0.001) in terms of educational level. More older respondents (78% within the ≥ 58 y group; 64% within the ≥ 46 y group) had a high level of education (more than secondary school), as compared to middle or low levels.
