# Supplementary material for: From simple to even simpler, but not too simple: a head-to-head comparison of the Better-Worse and Drop-Down methods for measuring patient health status
Source: BMC Med Res Methodol. 2023 Dec 16;23:299. doi: 10.1186/s12874-023-02119-9 (PMC10725035; doi:10.1186/s12874-023-02119-9)
Supplement: Supplementary file 4 — Additional file 4: Table A4. Characteristics of the total sample and of the sample excluding patients who reported full health state. [file 12874_2023_2119_MOESM4_ESM.docx]

Additional file 4

**Table A4**

Characteristics of the total sample and of the sample excluding patients who reported full health state

| **Characteristics** | **Total sample (1,927)** | **Excluding full health state (1389)** |
| --- | --- | --- |
| Gender, N (%) | 1,920 (100) | 1383 (100) |
| Female | 1,023 (53) | 802 (58) |
| Male | 897 (47) | 581 (42) |
|  |  |  |
| Age (year), N (%) | 1,921 (100) | 1383 (100) |
| 18–27 | 343 (18) | 256 (19) |
| 28–37 | 361 (19) | 256 (19) |
| 38–47 | 315 (16) | 210 (15) |
| 48–57 | 303 (16) | 239 (17) |
| 58–67 | 331 (17) | 244 (18) |
| 68–77 | 218 (11) | 143 (10) |
| ≥ 78 | 50 (3) | 35 (3) |
|  |  |  |
| Ethnicity, N (%) | 1,883 (98) | 1357 (98) |
| Asian/Asian-American | 85 (5) | 55 (4) |
| Black/African-American | 192 (10) | 114 (8) |
| Hispanic or Latino American | 113 (6) | 92 (7) |
| Native American/Inuit/Alaskan | 31 (2) | 20 (2) |
| Native Hawaiian/Pacific Islander | 7 (0) | 3 (0) |
| White American/Caucasian | 1,429 (76) | 1053 (77) |
| Other | 26 (1) | 20 (1) |
|  |  |  |
| Education, N (%) | 1232 (64) | 847 (61) |
| More than secondary school | 995 (52) | 663 (48) |
| Secondary school graduate | 204 (11) | 160 (12) |
| Less than secondary school | 33 (2) | 24 (2) |
|  |  |  |
