# Supplementary material for: From simple to even simpler, but not too simple: a head-to-head comparison of the Better-Worse and Drop-Down methods for measuring patient health status
Source: BMC Med Res Methodol. 2023 Dec 16;23:299. doi: 10.1186/s12874-023-02119-9 (PMC10725035; doi:10.1186/s12874-023-02119-9)
Supplement: Supplementary file 5 — Additional file 5: Table A5. Order effects (two study arms) on the difficulty of the BW and DD methods. [file 12874_2023_2119_MOESM5_ESM.docx]

Additional file 5

**Table A5**

Order effects (two study arms) on the difficulty of the BW and DD methods

| **Study arms** | **Difficulty of the BW method** | | **Difficulty of the DD method** | | **Which method do you find easier** | | |
| --- | --- | --- | --- | --- | --- | --- | --- |
|  | Mean scores*  (SD) | P value | Mean scores*  (SD) | P value | N (%) | | P value |
|  |  |  |  |  | BW | DD |  |
| **Total sample** | 30 (26) |  | 29 (26) |  | 1036 (54) | 877 (46) |  |
|  |  |  |  |  |  |  |  |
| **Study arms (N)** |  | 0.812^II^ |  | 0.318^II^ |  |  | 0.359^I^ |
| Study I (970) | 30 (26) |  | 28 (26) |  | 531 (55) | 431 (45) |  |
| Study II (957) | 30 (26) |  | 29 (26) |  | 505 (53) | 446 (47) |  |

I: Fisher exact test, II: T-test

* Scores for rating the difficulty of the BW/DD methods ranged from 0 to 100, with 0 indicating not difficult at all and 100 indicating the greatest possible difficulty.
