# Supplementary material for: From simple to even simpler, but not too simple: a head-to-head comparison of the Better-Worse and Drop-Down methods for measuring patient health status
Source: BMC Med Res Methodol. 2023 Dec 16;23:299. doi: 10.1186/s12874-023-02119-9 (PMC10725035; doi:10.1186/s12874-023-02119-9)
Supplement: Supplementary file 6 — Additional file 6: Table A6. Completion time (seconds) for the Better-Worse (BW) and Drop-Down (DD) methods. [file 12874_2023_2119_MOESM6_ESM.docx]

Additional file 6

**Table A6**

Completion time (seconds) for the Better-Worse (BW) and Drop-Down (DD) methods

| **Methods** | | **No. of respondents** | **Mean (SD)** | **95% Conf. interval** | **P value*** |
| --- | --- | --- | --- | --- | --- |
| All Drop-Downs^a^ | 1384 | | 99 (206) | [88, 110] | 0.008 |
| Better-Worse | 1384 | | 80 (183) | [70, 89] |  |
|  |  | |  |  |  |
| 5 Drop-Downs^b^ | 906 | | 109 (247) | [93, 125] | 0.028 |
| Better-Worse | 906 | | 85 (219) | [70, 99] |  |

* Paired T-test.

a: All respondents who did different times (1 to 5) of DD were counted.

b: Only respondents who did 5 times of DD were counted.
