# Supplementary material for: From simple to even simpler, but not too simple: a head-to-head comparison of the Better-Worse and Drop-Down methods for measuring patient health status
Source: BMC Med Res Methodol. 2023 Dec 16;23:299. doi: 10.1186/s12874-023-02119-9 (PMC10725035; doi:10.1186/s12874-023-02119-9)
Supplement: Supplementary file 7 — Additional file 7: Table A7. Impact of sociodemographic factors on the assessment of the difficulty of the BW and the DD methods. [file 12874_2023_2119_MOESM7_ESM.docx]

Additional file 7

**Table A7**

Impact of sociodemographic factors on the assessment of the difficulty of the BW and the DD methods

| **Sociodemographic factors** | **Difficulty of the BW method** | | **Difficulty of the DD method** | | **Which method do you find easier** | | |
| --- | --- | --- | --- | --- | --- | --- | --- |
|  | Mean scores*  (SD) | P value | Mean scores*  (SD) | P value | N (%) | | P value |
|  |  |  |  |  | BW | DD |  |
| **Gender** |  | 0.532^II^ |  | 0.853^II^ |  |  | 0.053^I^ |
| Females | 31 (25) |  | 28 (25) |  | 530 (52) | 489 (56) |  |
| Males | 30 (26) |  | 29 (27) |  | 501(48) | 386 (44) |  |
| **Age (y), group** |  |  |  |  |  |  |  |
| Divided by each decade |  | 0.019 ^III^ |  | 0.004 ^III^ |  |  | 0.023^I^ |
| 18-27 | 31 (24) |  | 31 (25) |  | 193 (57) | 146 (43) |  |
| 28-37 | 30 (25) |  | 28 (25) |  | 222 (62) | 138 (38) |  |
| 38-47 | 32 (26) |  | 29 (26) |  | 160 (51) | 154 (49) |  |
| 48-57 | 34 (27) |  | 32 (28) |  | 155 (52) | 143 (48) |  |
| 58-67 | 27 (26) |  | 26 (26) |  | 170 (52) | 160 (48) |  |
| 68-77 | 30 (27) |  | 27 (28) |  | 110 (50) | 108 (50) |  |
| ≥ 78 | 23 (22) |  | 20 (22) |  | 22 (46) | 16 (54) |  |
| Divided by 58-year^a^ |  | 0.008^Ii^ |  | 0.001^Ii^ |  |  | 0.042^I^ |
| 18-57 | 31 (26) |  | 30 (26) |  | 730 (56) | 581 (44) |  |
| ≥ 58 | 28 (26) |  | 26 (26) |  | 302 (51) | 294 (49) |  |
| Divide by 46-year^a^ |  | 0.994^Ii^ |  | 0.241^Ii^ |  |  | 0.004^I^ |
| 18-45 | 30 (25) |  | 29 (25) |  | 539 (58) | 398 (42) |  |
| ≥ 46 | 30 (27) |  | 28 (26) |  | 493 (51) | 477 (49) |  |
| **Education** |  | 0.346 ^III^ |  | 0.459 ^III^ |  |  | 0.991^I^ |
| More than secondary school | 28 (27) |  | 26 (26) |  | 539 (55) | 449 (45) |  |
| Secondary school graduate | 25 (22) |  | 24 (24) |  | 111 (55) | 91 (45) |  |
| Less than secondary school | 29 (24) |  | 30 (24) |  | 18 (56) | 14 (44) |  |

I: Fisher exact test, II: T-test, III: ANOVA test

* Scores for rating the difficulty of the BW/DD method ranged from 0 to 100, with 0 indicating not difficult at all and 100 indicating the greatest possible difficulty.

a: The 58-year age break is based on the values of seven age groups. The values for the three older age groups (58–67 years, 68–77 years, ≥ 78 years) were clearly larger than those for the younger age groups.

b: The 46-year age break is based on median age.
