# Supplementary material for: From simple to even simpler, but not too simple: a head-to-head comparison of the Better-Worse and Drop-Down methods for measuring patient health status
Source: BMC Med Res Methodol. 2023 Dec 16;23:299. doi: 10.1186/s12874-023-02119-9 (PMC10725035; doi:10.1186/s12874-023-02119-9)
Supplement: Supplementary file 8 — Additional file 8: Table A8. Correlation between age and difficulty scores for the BW and DD methods. [file 12874_2023_2119_MOESM8_ESM.docx]

Additional file 8

**Table A8**
Correlation between age and difficulty scores for the BW and DD methods

|  | **Pearson correlation**  **coefficient** | **P value** |
| --- | --- | --- |
| Difficulty scores for BW (N=1897) | -0.028 | 0.227 |
| Difficulty scores for DD (N=1903) | -0.057 | 0.013 |

Both the BW and the DD method showed very weak decreasing linear relationships between age and scores for the relative difficulty of the two methods. The Pearson correlation coefficient for the BW method, -0.028, was not statistically significant (P = 0.227). The Pearson correlation coefficient for the DD method, -0.057, was statistically significant (P = 0.013).

**Figure A8**
Scatter plot between age and difficulty scores* for the BW and DD methods

* Scores for rating the difficulty of BW/DD methods ranged from 0 to 100, with 0 indicating not difficult at all and 100 indicating the greatest possible difficulty.
