# Supplementary material for: From simple to even simpler, but not too simple: a head-to-head comparison of the Better-Worse and Drop-Down methods for measuring patient health status
Source: BMC Med Res Methodol. 2023 Dec 16;23:299. doi: 10.1186/s12874-023-02119-9 (PMC10725035; doi:10.1186/s12874-023-02119-9)
Supplement: Supplementary file 9 — Additional file 9: Table A9. Correlation between Age and completion time (second) for the BW and the DD methods (N=1384). [file 12874_2023_2119_MOESM9_ESM.docx]

Additional file 9

**Table A9**

Correlation between Age and completion time (second) for the BW and the DD methods (N=1384)

|  | **Pearson correlation**  **coefficient** | **P value** |
| --- | --- | --- |
| Completion time BW | 0.073 | 0.007 |
| Completion time DD | 0.057 | 0.036 |

Both the BW and the DD methods showed very weak increasing linear relationships between age and completion time.

**Figure A9**

Scatter plot between age and completion time for the BW and the DD methods
