# Supplementary material for: From simple to even simpler, but not too simple: a head-to-head comparison of the Better-Worse and Drop-Down methods for measuring patient health status
Source: BMC Med Res Methodol. 2023 Dec 16;23:299. doi: 10.1186/s12874-023-02119-9 (PMC10725035; doi:10.1186/s12874-023-02119-9)

Additional file 10

**Table A10**

The number of observations of each level of CS-Base items by BW and DD methods (N=1927)

| **CS-Base items and levels N (%)** | **BW method** | **DD method** |
| --- | --- | --- |
| Mobility |  |  |
| No problems | 1425 (74) | 1430 (74) |
| Some problems | 377 (20) | 364 (19) |
| Moderate problems | 93 (5) | 96 (5) |
| Severe problems | 32 (2) | 37 (2) |
| Vision |  |  |
| Good | 1540 (80) | 1540 (80) |
| Impaired | 339 (18) | 332 (17) |
| Poor | 43 (2) | 49 (3) |
| Blind | 5 (0) | 6 (0) |
| Hearing |  |  |
| Good | 1153 (60) | 1131 (59) |
| Impaired | 645 (33) | 671 (35) |
| Poor | 122 (6) | 118 (6) |
| Deaf | 7 (0) | 7 (0) |
| Cognition |  |  |
| No problems | 1622 (84) | 1625 (84) |
| Some problems | 253 (13) | 255 (13) |
| Moderate problems | 42 (2) | 38 (2) |
| Severe problems | 10 (1) | 9 (0) |
| Mood |  |  |
| Good | 1222 (63) | 1232 (64) |
| Slightly sad | 448 (23) | 441 (23) |
| Sad | 171 (9) | 175 (9) |
| Dark | 86 (4) | 79 (4) |
| Anxiety |  |  |
| Not anxious | 977 (51) | 1012 (53) |
| Slightly anxious | 523 (27) | 503 (26) |
| Anxious | 281 (15) | 265 (14) |
| Highly anxious | 146 (8) | 147 (8) |
| Pain |  |  |
| No | 852 (44) | 878 (46) |
| A little | 659 (34) | 630 (33) |
| Moderate | 318 (17) | 323 (17) |
| Severe | 98 (5) | 96 (5) |
| Fatigue |  |  |
| Not tired | 857 (44) | 895 (47) |
| A little tired | 630 (33) | 606 (31) |
| Quite tired | 338 (18) | 332 (17) |
| Very tired | 102 (5) | 94 (5) |
| Social function |  |  |
| No problems | 1297 (67) | 1335 (69) |
| Some problems | 427 (22) | 393 (20) |
| Moderate problems | 139 (7) | 141 (7) |
| Severe problems | 64 (3) | 58 (3) |
| Daily activity |  |  |
| No problems | 1285 (67) | 1305 (68) |
| Some problems | 484 (25) | 462 (24) |
| Moderate problems | 121 (6) | 124 (6) |
| Severe problems | 37 (2) | 36 (2) |
| Self-esteem |  |  |
| Strong | 1049 (54) | 1039 (54) |
| Good | 409 (21) | 416 (22) |
| Low | 360 (19) | 354 (18) |
| Very weak | 109 (6) | 118 (6) |
| Independence |  |  |
| Independent | 1479 (77) | 1500 (78) |
| Somewhat dependent | 339 (18) | 330 (17) |
| Largely dependence | 85 (4) | 77 (4) |
| Fully dependent | 24 (1) | 20 (1) |

In DD method, although “vision” and “hearing” has less responses in Task 1 (table above) than “cognition”, they got more responses in task 2 and after generating postulated health states. The final number of observations on level-4 of “vision” and “hearing” was 43 and 50, while it was the least for “cognition” (37). This can explain why level-4 of cognition showed a largest confidence interval.


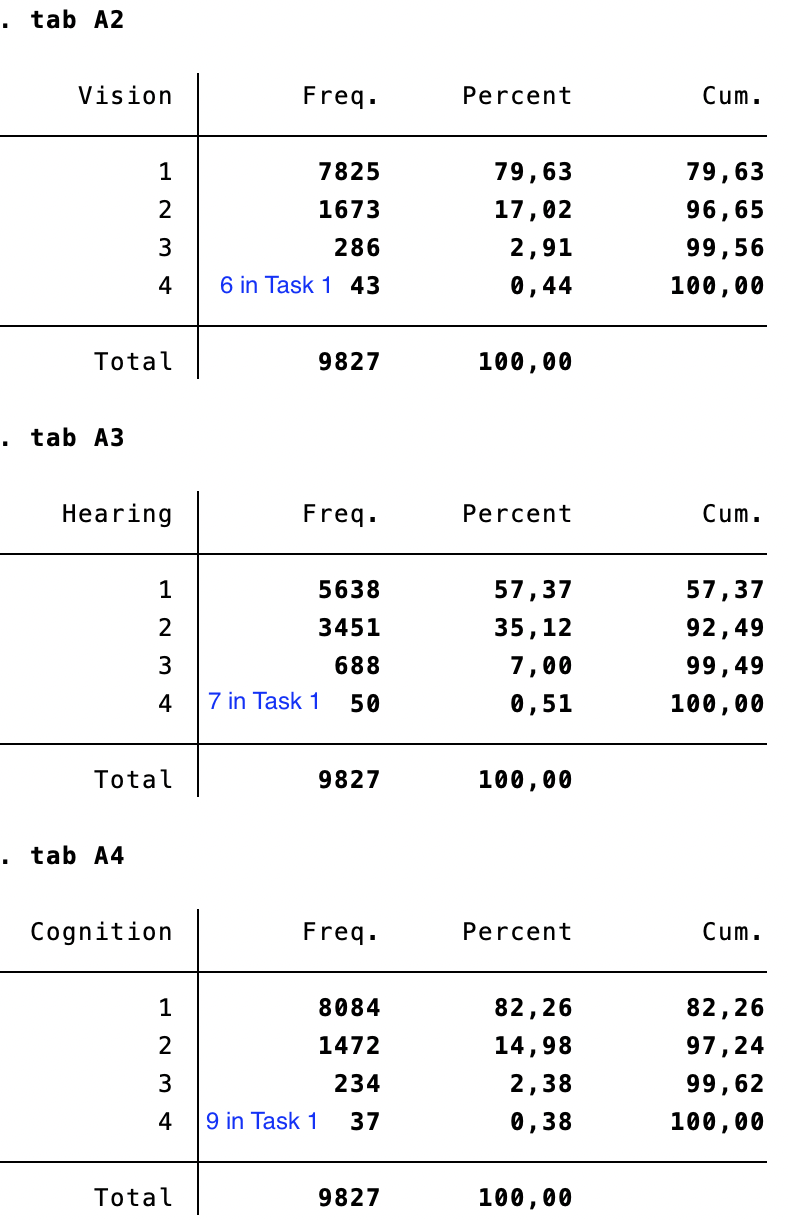

Supplement: Supplementary file 10 — Additional file 10: Table A10. The number of observations of each level of CS-Base items by BW and DD methods (N=1927). [file 12874_2023_2119_MOESM10_ESM.docx]
