# Supplementary material for: From simple to even simpler, but not too simple: a head-to-head comparison of the Better-Worse and Drop-Down methods for measuring patient health status
Source: BMC Med Res Methodol. 2023 Dec 16;23:299. doi: 10.1186/s12874-023-02119-9 (PMC10725035; doi:10.1186/s12874-023-02119-9)
Supplement: Supplementary file 11 — Additional file 11: Figure A11. Scatter plot of BW and DD coefficients. [file 12874_2023_2119_MOESM11_ESM.docx]

Additional file 11

**Figure A11**

Scatter plot of BW and DD coefficients
